# Exploring an Association between Body Mass Index and Oral Health—A Scoping Review

**DOI:** 10.3390/diagnostics13050902

**Published:** 2023-02-27

**Authors:** Rakhi Issrani, Jagat Reddy, Alzarea K. Bader, Raghad Fayez H. Albalawi, Ebtehal Dhyab M. Alserhani, Danah Sultan R. Alruwaili, Gharam Radhi A. Alanazi, Naif Sultan R. Alruwaili, Mohammed Ghazi Sghaireen, Krishna Rao

**Affiliations:** 1 Department of Preventive Dentistry, College of Dentistry, Jouf University, Sakaka 72388, Saudi Arabia; 2 Department of Oral Medicine & Radiology, Indira Gandhi Institute of Dental Sciences, SBV University, Puducherry 607402, India; 3 Department of Prosthetic Dental Sciences, College of Dentistry, Jouf University, Sakaka 72388, Saudi Arabia; 4 College of Dentistry, Jouf University, Sakaka 72388, Saudi Arabia; 5 Fayez Dental Complex, Qurayyat 77453, Saudi Arabia

**Keywords:** basal metabolic index, dental caries, inflammation, obesity, oral health, periodontitis

## Abstract

Background: Basal metabolic index (BMI) is a unique anthropometric indicator used to define the relative amount of body fat on an individual’s frame. There are many diseases and conditions associated with obesity and underweight. Recent research trials suggest that there is a significant association between oral health indicators and BMI as both are attributed to common risk factors such as dietary, genetic, socioeconomic, and lifestyle issues. Objectives: The main objective of this review paper is to emphasize the association between BMI and oral health with available literature evidence. Methodology: A literature search was conducted using multiple databases comprising of MEDLINE (via PubMed), EMBASE, and Web of Science. The terms used for the search were “body mass index”, “periodontitis”, “dental caries”, and “tooth loss”. Results: In total, 2839 articles were obtained from the analysis of the databases. Unrelated articles from the available full text of 1135 articles were excluded. The main reasons for excluding the articles were: they were dietary guidelines and policy statements. A total of 66 studies were finally included in the review. Conclusion: The presence of dental caries, periodontitis and tooth loss may be associated with a higher BMI or obesity, whereas, improved oral health might be associated with lower BMI. Promoting general and oral health should be a hand in hand feature, as common risk factors can be embattled.

## 1. Introduction

In the modern era, the most challenging community health issue is the growing number of overweight and obese individuals [1]. A recent study showed that between 1980 and 2014, the prevalence of obesity and overweight increased globally [2]. It is interesting to note that the WHO estimated that almost 2.5 billion persons over the age of 18 suffer from obesity and overweight in 2014 [2]. This is around a third of the entire world’s population [2,3]. Between nations and regions within nations, the incidence varies greatly. Men are more likely to be overweight than women, while women are more likely to be obese [3]. In addition, obesity is currently ranked as the fifth biggest cause of death globally and the risk of death increases by 20–40% in overweight individuals, and it escalates to 200–400% in obese individuals. Every year, obesity and overweight cause the death of around 3.4 million people [2,4,5].

Obesity has been linked to a number of illnesses, including heart disease, musculoskeletal problems, hypertension, type 2 diabetes, and several cancers such as breast, prostate, liver, and colon cancer [6,7]. This adds to the burden of medical costs for obese individuals that are found to be 30% greater than for normal-weight peers [8]. Despite the fact that certain people are genetically predisposed to obesity, recent rapid environmental changes, freely available “high fat food,” and a decline in physical activity have caused obesity rates to rise not only in industrialized but also in developing nations around the world [9].

Body mass index (BMI) is one particular anthropometric measure that is used to describe the relative amount of body fat in an individual’s frame [10]. BMI was introduced by a Belgian astronomer, mathematician, and statistician named Adolphe Quetelet in 1835 and it was previously referred to as the Quetelet’s index [11]. It has always been considered as a simple method for analysis of the nutritional status and has been in use since the mid-19th century [10,12]. It is determined by multiplying the weight in kilograms by the square of the height in meters, and is represented in kilograms per square meter (kg/m^2^) [10,13]. According to the WHO, BMI can be divided into four categories for all adult age groups: underweight, normal, overweight, and obese [14]. The classification of overweight and obesity provided by the National Institutes of Health is shown in Table 1 [15].

It has been found that people with BMI in the normal range typically have a quick metabolism, as opposed to obese people, who typically have a slower resting metabolic rate. This is because obese people tend to have more muscle mass, which increases resting metabolic rate [13]. Both fat and fat-free mass make up an obese person’s body mass, and since fat mass does not contribute much to metabolism, their metabolic rate is low [16].

Dental caries, periodontitis, and tooth loss are typical illnesses caused by poor oral health [17]. As both are linked to shared risk factors such diet (consumption of sugary drinks, and snacks), hereditary, socioeconomic, and lifestyle changes, recent research studies reveal that there is a considerable correlation between oral health indicators and BMI [18]. The occurrence of periodontitis, dental caries, and tooth loss may be linked to higher BMI, according to a new study that included individuals from the national health screening cohort in Korea from 2009 to 2010 [17]. It is believed that inflammation plays a significant role in the relationship between oral disorders and obesity [17].

The main objective of this review paper is to emphasize the association between BMI and oral health with available literature evidence. The purpose of this paper is to highlight the mutual risk factors associated with obesity and dental caries, periodontitis, and tooth loss.

## 2. Materials and Methods

This review is in accordance with the Preferred Reporting Items for Systematic Review and Meta-analysis (PRISMA) 2020 Statement in order to maintain a codified organization of the study [19] (available as a supplemental material). The search databases included MEDLINE (via PubMed), EMBASE, and Web of Science. The terms used for the search were “body mass index”, “periodontitis”, “dental caries”, and “tooth loss”. Further searches were performed in the reference lists of relevant studies and in literature reviews dealing with the topic of interest.

Considering the eligibility criteria, articles relevant to the topic of BMI and oral health indicators were evaluated as suitable for inclusion in this review. Only papers published within the last 5 years (30 September 2017 to 30 September 2022) and written in English language were considered. Additionally, only original papers were considered. Interim reports, abstracts, letters, short communications, and chapters in textbooks were discarded.

All the studies resulting from the search strategies were imported into an Endnote library and duplicates were removed. Two reviewers (first and second author) independently assessed the records (title and abstract), selecting the articles that met the eligibility criteria. Any type of disagreement was resolved by consulting a third independent reviewer (third author). After this screening, the records selected were analyzed in their full-text version, and two other reviewers (first and second authors) independently assessed whether they should be included in the review. In case of disagreement, a third author was consulted (third author). The same two reviewers carried out the extraction of the data in a standardized data form.

**Figure 1:**
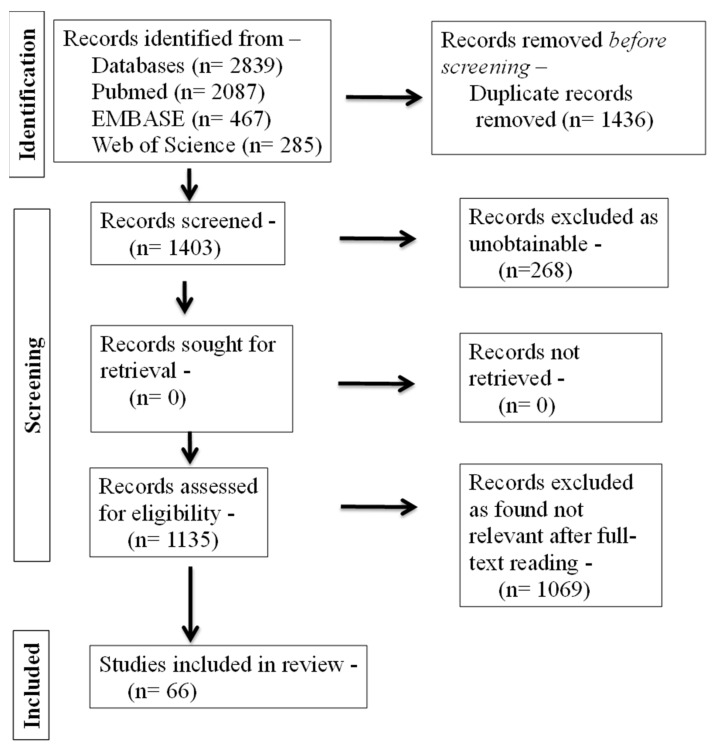
PRISMA flowchart of study selection process.

The PRISMA flow diagram (Figure 1) was used to report the included articles according to the eligibility criteria and those excluded during the study selection process.

## 3. Results

In total, 2839 articles were obtained from the analysis of the databases, adopting the search strategy described in Section 2. Unrelated articles from the available full text of 1135 articles were excluded. The main reasons for excluding the articles were: they were dietary guidelines and policy statements. A total of 66 studies were finally included in the review.

## 4. Discussion

This paper aimed at exploring an association between BMI and oral health. This review will contribute toward a better understanding of risk factors related to obesity and oral diseases. There exists a “bidirectional relationship” between oral and systemic health [20]. An individual’s overall health is influenced by their oral health that is related to preserving the health of the perioral tissues, craniofacial complex, periodontal tissues, and dentition [20]. Urbanization and modernization, along with unfavorable dietary changes aimed at increasing consumption of fat and sugar while reducing intake of roughage, have all contributed to the development of unhealthy eating habits. The oral health suffers as a result, and the BMI rises [21]. Younger people have been seen to consume high-density foods, which replace fiber-rich diets with fat- and sugar-rich foods that have a considerably higher level of preservatives and low nutritional value [22].

Dental caries, which affects between 60% and 90% of school children and the majority of adults worldwide, is the most common non-communicable disease and has long been regarded as the greatest global oral health burden [23,24]. Despite the availability of advanced and innovative therapeutic approaches, it remains a public health issue [25]. Dental caries is a term used to describe an irreversible microbiological condition that affects the calcified tissues of the teeth. It is characterized by demineralization of the inorganic portion and destruction of the organic section of the tooth, which frequently results in cavitation [25]. Although it has a complex etiology and pathobiology, nutrition, along with dental hygiene, saliva, and oral flora, have a key influence in the initiation and advancement of caries [26]. Additionally, it has been noted that the high prevalence of dental caries may be due to poor oral health awareness among the population [27].

Regarding the relationship between an individual’s BMI and the state of their dental health and hygiene, the literature has conflicting findings [28]. While some studies claimed there was no connection at all between BMI and dental caries [29,30] other studies demonstrated a significant association between overweight/obesity and high caries incidence [31,32]. Only one study with a high level of evidence, according to a systematic review done in 2006, found a direct and significant link between dental caries and obesity [33]. A meta-analysis conducted by Chen et al. highlighted that obese group of individuals had more caries than the normal-weight group in their primary teeth. Caries incidence was significantly higher among the overweight and obese children in high-income countries, but not in low- and middle-income countries. This could be attributed to the lifestyle and dietary habits [34]. In their various research, Willershausen et al. [29], Thippeswamy et al. [30], Jouhar et al. [35], Modeer et al. [36], and Khattak et al. [37] have found that obese subjects exhibit a higher number of decayed surfaces in comparison to non-obese subjects. The majority of children with caries, according to Haliti F et al., were in the healthy weight and obesity group, followed by the overweight group and the underweight group [38]. On the other hand, contradictory results were highlighted in a study conducted by Idrees et al. [4], Sede et al. [31], and Kim et al. [39] where BMI was not associated with dental caries. Similarly, Abdellatif et al. found no association between BMI categories and mean decayed missing filled index. It was concluded that BMI may be considered as a related factor and undoubtedly not a secluded risk factor for dental caries [40]. According to Americano et al., underweight or malnutrition may significantly affect tooth development, leading to poorly developed or hypomineralized dental enamel, which is a known risk factor for the occurrence of caries [41]. According to another theory, severe dental caries can lower BMI because it makes it difficult to correctly chew or bite food, whereas early or immature caries have no impact on nutrition intake [42].

Obesity and caries are both largely influenced by diet, which includes consuming too many refined carbohydrates, particularly sugar in its refined form [43]. Additionally, frequent snacking has been connected to obesity and other chronic conditions. According to a study by Alswat et al., younger people with high BMI, who drank sugary drinks and led sedentary lifestyles had a higher incidence of caries [44]. The findings, according to the authors, could be explained by the fact that obese and overweight people spend more time online using social media, which suggests that a combination of a sedentary lifestyle and frequent snacking may have contributed to the weight increase. Additionally, Aljefree et al. found in their study that fewer than half of the participants in the survey consumed snacks once per day, which decreased the likelihood of gaining weight [45]. However, according to Aljuraiban et al., frequent meals have been linked statistically significantly to reduced BMI [46]. It has been identified that reducing the frequency of meals per day may have an adverse effect on appetite control [47]. There is evidence that an increase in meal frequency along with hormonal and nutritional signals can suppress appetite [48]. This results in decreased energy levels and delayed stomach emptying, which reduces the feeling of hunger [49]. Thus, there is debate concerning the relationship between a patient’s weight and their dental health state [28]. This controversy is partially related to the nature of the published studies, which primarily sampled children or teenagers under the age of 18, with only a small number of studies testing this link among adult individuals [33,50,51]. In our perspective, variations in genetic predisposition to caries and obesity, lifestyle, and dietary habits, which are particular to each community and demographic, could be the cause of the disparity and contradiction of results among the research.

**Figure 2:**
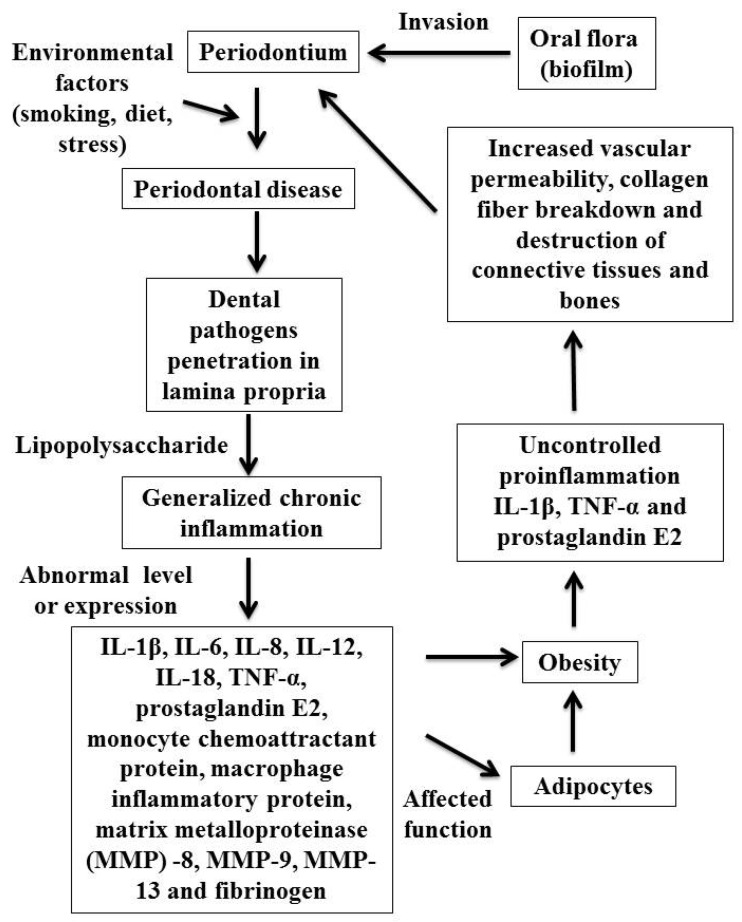
Probable connections between periodontal diseases and obesity.

Periodontal disease has been considered as one of the extremely prevalent condition globally and is represented as a leading public health dilemma for both developing and developed society [7]. Periodontitis is defined as an inflammatory disease of the supporting tissues of teeth caused by specific microorganisms or groups of specific microorganisms, resulting in progressive destruction of the periodontal ligament and alveolar bone with periodontal pocket formation, gingival recession, or both [15]. Age, smoking, oral hygiene, socioeconomic status, genetics, race, gender, psychosocial stress, osteopenia, osteoporosis, and various other systemic diseases such as type 2 diabetes mellitus and cardiovascular disease are all risk factors for periodontal diseases. This means that periodontitis does not only develop as a result of plaque deposition but is also associated with various host factors that could change the outcome of the condition [7]. There are numerous mechanisms that could account for a link between obesity and periodontitis [52]. Young people who are overweight, in particular, have poor eating habits that include eating too much sugar and fat and not enough micronutrients. Such dietary habits may affect periodontal tissues. Early -life excessive weight gain, which is frequently linked to higher levels of stress, may also contribute to the promotion of periodontal disease. Diversification in the oral environment or mild chronic inflammation may be caused by an abundance of adipose tissue. In obese people, adipokines—a class of bioactive molecules-up to 50 are released by adipose cells. Leptin, adipocytokines, and adiponectin are examples of proteins that resemble hormones, whereas tumor necrosis factor (TNF) and interleukins (IL) are examples of classical cytokines [53]. TNF-α, IL-1, and IL-6 are among the pro-inflammatory cytokines produced as a result of the increased macrophages and adipocytes. Increased production of these pro-inflammatory cytokines effects the host susceptibility towards the evolution and advancement of periodontal diseases since the release of inflammatory cytokines is directly associated with a higher vulnerability to bacterial infection [54]. Insulin resistance is another potential link between obesity and periodontal disease. Dietary-free fatty acids contribute to both insulin resistance and obesity by causing abolition of beta cells of pancreas. Insulin resistance thus contributes to a generalized hyperinflammatory condition that affects periodontal tissue [53,54]. Figure 2 shows a probable connection between periodontal diseases and obesity [54].

Numerous research have found an association between greater BMI and periodontitis, while a small number have found no such association. Previous research by Deshpande NC et al. [7], Kim et a. [39], Al-Zahrani et al. [55], and Gulati et al. [56] found a substantial correlation between periodontitis and obesity. In a same manner, Cetin et al. discovered a statistical relationship between BMI and clinical attachment loss, probing pocket depth, plaque index, stage and grade of periodontitis, and the number of remaining teeth. It was concluded that BMI increases the risk of developing stages III and IV of periodontitis [57]. A study conducted by Chen et al. on a large population-based dataset in Taiwan revealed that patients who were obese had a 1.12-fold higher chance of developing chronic periodontitis than those without obesity [58]. Similarly, in a meta-analysis of previous cross-sectional studies, periodontitis was strongly associated with increased BMI [59]. In the study conducted by Chang Y et al., periodontitis was independently associated with BMI regardless of other oral hygiene indicators [17]. The results of a study by Sede et al., on the other hand, showed no association between BMI and periodontitis, but they did identify a significant correlation between the gingival bleeding index and BMI [31]. The majority of obesity research conducted so far have focused on adults and elderly people. An analytical review of Suvan et al. noticed a heterogeneity among various studies, where higher odds of periodontitis was noted among obese individual but, no studies where identified among young adults that have evaluated whether obesity directly causes periodontitis [10].

Furthermore, it has been discovered that BMI is related to frequency of tooth brushing. According to the study by Alam BF et al., there was a statistically significant correlation between the majority of participants who cleaned their teeth twice a day and had varied BMIs [60]. A recent general population-based longitudinal study of 4537 participants in Japan demonstrated that a low frequency of tooth brushing (≤1 time/day) was associated with the occurrence of obesity [61]. This is due to the fact that poor oral hygiene, in addition to triggering an inflammatory response within the oral cavity, also results in an increase in C reactive protein levels, which has been related to obesity [62]. Brushing with fluoride toothpaste aids in removing the bacterial biofilm that is present on tooth surfaces and is a major contributor to the development of various oral disorders, including dental caries, periodontal disease, and pulpal diseases [63]. Additionally, leptin-linked pathways, which regulate the equilibrium between energy and appetite, are likely to be responsible for the connection between teeth brushing and obesity. As a result, regular and proper tooth brushing can help to reduce appetite and lower the risk of obesity [62]. Therefore, it is crucial to highlight the significance of maintaining good oral hygiene because obesity and being overweight have a close relationship with periodontal disorders [60]. However, the study led by Chang et al. identified that individuals who brushed their teeth twice daily had lower BMI [17]. This difference could be attributed to the different study populations. It is worth noting that a statistically significant association was seen regarding using mouthwash as the most common aid with BMI in the study done by Jouhar et al. [35], Modeer et al. [36], and Alam BF et al. [60].

Tooth loss is the separation of a tooth from its supporting tissues as a result of trauma, caries, periodontal disease, a cracked tooth, endodontic failure, etc., [64]. Mastication, digestion, phonation, and aesthetics could all be significantly impacted, which could have an adverse effect on quality of life. A partially edentulous patient’s eating habits may change from fibrous and dry food to a more soft diet that is likely to be high in fat and carbohydrates and deficient in vitamins and minerals. This change in diet may increase a person’s risk of obesity or higher BMI [65]. Contrarily, it is also evident that edentulous patients find it difficult to chew foods with a rough texture; as a result, these people modify their diets to recompense for the loss of oral function. These individuals have a lower BMI or are more likely to be underweight due to inadequate nutrition [66].

A few studies have shown that those with a higher BMI had decreased salivary flow, which could accelerate up the onset of caries and lead to tooth loss. This may be related to generalized inflammation of low intensity, which releases cytokines that promote inflammation. The hypothalamic-pituitary-adrenal axis and cytokines have the ability to modulate the central nervous system activity and reduce salivary flow. Obesity is linked to low-grade chronic inflammation, which is a contributing factor in periodontitis, as noted in the literature. Presence of periodontitis may ultimately result in loss of teeth [67]. Sonoda et al. carried out a study to determine the relationship between eating speed and oral health status with obesity in Japanese working males. It was found that people who had more number of missing functional teeth and had severe periodontal disease were more likely to have a waist circumference over 90 cm. Therefore, it was established that the eating speed, the number of functional teeth missing, and severe periodontal disease are all independently related with a larger waist circumference or higher BMI [68]. Natrajan et al. investigated the association between BMI and missing posterior teeth in relation to age and socioeconomic status. People with severe tooth loss were shown to be more obese, while low socioeconomic group obese females had significantly higher tooth loss. No significant relation between age and obesity was found with regard to tooth loss [65]. In a Brazilian study of elderly individuals, tooth loss was significantly correlated with obesity [69]. In contrast to previously reported studies, Selvamani et al. examined the relationship between underweight and self-reported edentulism among older men and women. In elderly women, it was found that there was a strong positive correlation between underweight and edentulism. Underweight is associated with biological changes including early menopause. A higher likelihood of edentulousness exists in underweight women who experienced early menopause [70]. Song et al. used data from a nationally representative sample to find correlations between the number of natural teeth and BMI. The increased risk of tooth loss and those who were underweight were found to be statistically significantly associated [71]. The relationship between edentulism and reduced BMI may be explained by the person’s nutritional deficiencies. The weakened immune systems make them more susceptible to infectious diseases. It might also be attributable to inadequate masticatory function [72]. Sheiham A et al. found that people without teeth were significantly more likely to be underweight than those with 11 or more teeth [73]. It is evident that there is a complex association between BMI and oral health. A low BMI is easily explained by the presence of actual functional challenges that, in some circumstances, can prohibit normal eating. On the other hand, the relationship between poor oral health and obesity is probably related to the quality of the diet. There may even be an argument that the loss of teeth may have been the result of a poor quality diet in some instances. Interestingly, the strongest associations between BMI (both being underweight and obese) were where people had some teeth, but were not edentulous. Where there are a few poorly distributed teeth compared to none at all, it may be more challenging to provide adequate function and, consequently, a sufficient and varied diet. This is particularly difficult if only the maxillary teeth are present. Though it is not always the case, this may help to explain some of the observed associations [73].

### Limitations

This study did not take into account a variety of potential confounders, including socioeconomic and lifestyle characteristics, genetic predisposition, age, sex, stress, and smoking status related to body weight and oral health.

## 5. Conclusions

A potential risk factor for periodontal disease, dental caries, or tooth loss includes being overweight or obese. As common risk factors might be compromised, promoting general and oral health as well as physical activity should go hand in hand. A vital part of keeping a healthy body mass index is having a healthy, functional dentition combined with strict hygiene.

## Figures and Tables

**Table 1:** Categorization for BMI.

CLASS	BMI (in kg/m^2^)
Underweight	<18.5
Normal	18.5–24.9
Overweight	25.0–29.9
Obesity	
Class I	30.0–34.9
Class II	35.0–39.9
Class III	≥40

## Data Availability

The data set used in the current paper will be made available on request from Rakhi Issrani.
